# Evaluation of three APBI techniques under NSABP B‐39 guidelines

**DOI:** 10.1120/jacmp.v11i1.3021

**Published:** 2009-12-03

**Authors:** Daniel Scanderbeg, Catheryn Yashar, Greg White, Roger Rice, Todd Pawlicki

**Affiliations:** ^1^ Department of Radiation Oncology UC San Diego CA USA

**Keywords:** SAVI, MammoSite, 3D‐CRT, APBI, breast cancer

## Abstract

This work compares two accelerated partial breast irradiation modalities, MammoSite brachytherapy and three‐dimensional conformal radiotherapy (3D‐CRT), to a new method, strut‐adjusted volume implant (SAVI) brachytherapy, following NSABP B‐39 guidelines. A total of 21 patients treated at UC San Diego with the SAVI device were evaluated in this comparison. Nine of the 21 patients were eligible for all three modalities and were dosimetrically compared evaluating V90, V150, V200, total target volume, maximum skin, lung, and chestwall/rib dose. The target volumes (PTV_EVAL) differed with SAVI, having the least total volume at 59.9 cc vs. 71.5 cc and 351.6 cc for MammoSite and 3D‐CRT, respectively. The median V90, V150 and V200 for the three modalities were 97.7%, 25.0 cc, 10.4 cc (SAVI) vs. 97.6%, 23.9 cc, 5.0 cc (MammoSite) vs. 100% (V90 3D‐CRT). The maximum dose for SAVI, MammoSite, and 3D‐CRT, respectively, relative to the prescribed dose, for the lung: 80.0%, 150.0%, and 104.9%; for rib: 108.8%, 225.0%, and 114.7%; for skin: 75.0%, 135.0%, and 108.6%. Comparing modalities, PTV coverage varied between 97.6%–100.0% with more breast tissue covered by 3D‐CRT, as expected, given the differences between external beam and brachytherapy. The maximum lung, skin and rib doses were lowest for the SAVI, highlighting its ability to conform to exclude normal tissues. In offering partial breast radiation, the availability of a variety of techniques allows for maximal patient eligibility, and comparison of individual method pros and cons may guide the most appropriate choice for each patient.

PACS number: 87.53.Jw; 87.53.Kn; 87.55.D

## I. INTRODUCTION

The NSABP B‐39/RTOG 0413 trial is a phase III randomized trial that was initiated in March 2005 to study the equivalency between whole breast irradiation (WBI) and accelerated partial breast irradiation (APBI) with regard to locoregional control, overall survival, cosmetic results, and any treatment related symptoms. Typically, WBI irradiates the entire breast with a 6‐week course of treatment. This type of treatment has the drawback of excess irradiation of normal tissue as well as being inconvenient for the patient due to the long duration. APBI has emerged over the past decade as a viable option for radiotherapy following lumpectomy. APBI involves a shorter course of radiotherapy (~5days) delivered in larger doses per fraction to a more focused target volume.

The three treatment modalities offered under the APBI arm of the NSABP B‐39 study are interstitial brachytherapy, MammoSite brachytherapy and three‐dimensional conformal radiotherapy (3D‐CRT). As APBI has become increasingly more popular for early stage breast cancers, the number of available delivery techniques has increased. One of the new delivery modes is the Strut‐adjusted Volume Implant or SAVI (Cianna Medical, Aliso Viejo, CA). The SAVI is a kitchen whisk‐like device with a central catheter surrounded by several peripheral catheters. Each catheter can be loaded with the HDR source to shape dose to the planning target volume (PTV).

This paper compares two of the “traditional” modalities used in the NSABP B‐39 trial (namely, MammoSite brachytherapy and 3D‐CRT) to the SAVI modality. With the advent of new devices and their increased clinical use, a dosimetric comparison between modalities is essential for selecting the proper modality for a particular patient's treatment. A comparison of the techniques allows one to select the best treatment option for a patient based on the dosimetric capability of the device. There have been a few studies that compare APBI techniques.^(^ ^1^ ^–^ ^3^ ^)^ To our knowledge, none of these studies have included the SAVI device in the comparison.

## II. MATERIALS AND METHODS

### A. The MammoSite device and planning criteria

MammoSite breast brachytherapy has been in use for almost a decade.^(^ ^4^ ^–^ ^8^ ^)^ The device is a single‐entry applicator that has a central strut which can be loaded with the high‐dose rate (HDR) source in a centrally located position inside the balloon. Since the source has a single dwell position in the center of the device, the dose distribution is approximately spherical, thus leading to equal dosing around the lumpectomy cavity despite the shape of the planning target volume. The prescribed dose is 34 Gy in five days, treated twice daily. The planning criteria governing treatment as described in the NSABP B‐39 protocol are shown in Table 1.

**Table 1:** Dosimetric guidelines for MammoSite, per NSABP B‐39 protocol.

V90	>90%
V150	<50cc
V200	<10cc
Skin Dose	<145% of prescribed dose

### B. 3D‐CRT and planning criteria

3D‐CRT therapy offers a non‐invasive radiation treatment option for patients with an accelerated course of therapy over five days. The prescribed dose is 38.5 Gy in five days, treated twice daily. Typical 3D‐CRT plans consist of 3–5 noncoplanar fields with no beams directed towards the heart, lung or contralateral breast.^(9)^ 3D‐CRT offers a minimally invasive technique compared to any of the brachytherapy modalities, but at the expense of irradiating more normal tissue. Planning criteria as specified in the NSABP B‐39 protocol are shown in Table 2.

**Table 2:** Dosimetric guidelines for 3D‐CRT, per NSABP B‐39 protocol.

Uninvolved Normal Breast	<60% of whole breast ref volume receives ≥50% of prescribed dose; <35% ref volume should receive prescribed dose
Contralateral Breast	<3% of prescribed dose to any point
Ipsilateral Lung	<15% of lung can receive 30% of prescribed dose
Contralateral Lung	<15% of lung can receive 5% of prescribed dose
Heart (rt‐sided lesions)	<5% of heart should receive 5% of prescribed dose
Heart (lt‐sided lesions)	Volume of heart getting 5% of dose (V5) should be less than 40%
Thyroid	Maximum point dose of 3% of prescribed dose
V90	≥90%
Critical Normal Tissue DVHs	Within 5% specified value
Maximum Dose	<120% of prescribed dose

### C. The SAVI device and planning criteria

**Figure 1:**
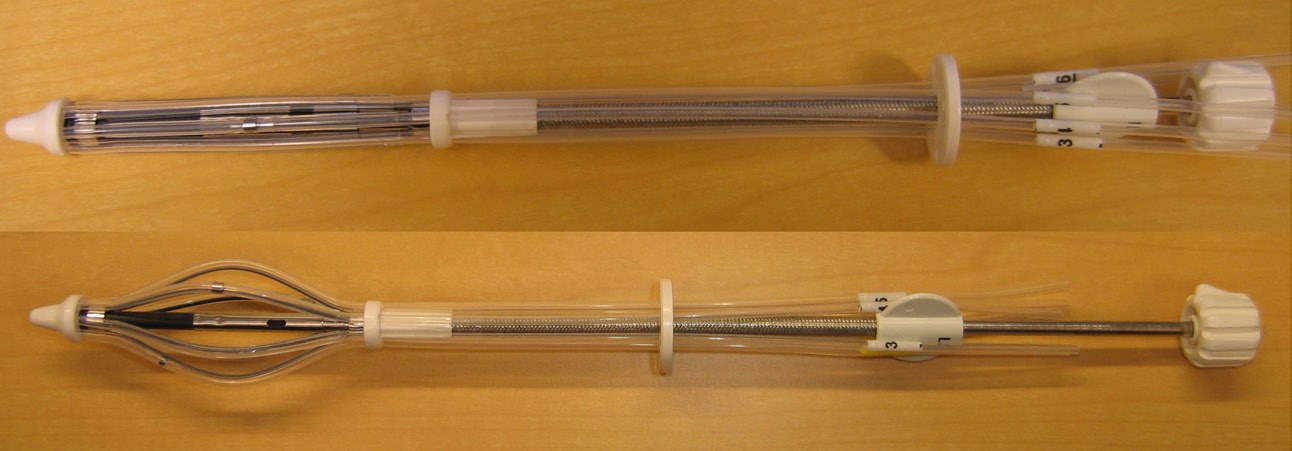
SAVI APBI brachytherapy device: a) collapsed for insertion and removal; b) expanded showing central strut surrounded by peripheral struts.

The SAVI breast brachytherapy device (Fig. 1) is one of several new devices on the market that combines the simplicity of a single‐entry applicator with multiple peripheral catheters that open to lie against the lumpectomy cavity walls. The device has a central strut and multiple peripheral struts that can be differentially loaded with the HDR source. The prescribed dose is the same as MammoSite – 34 Gy in 5 days, treated twice daily. The planning criteria used are shown in Table 3. The three criteria were taken from the NSABP B‐39 protocol for MammoSite brachytherapy, with V200 taken from interstitial criteria. Wazer et al.^(10)^ has shown that fat necrosis and poor cosmetic outcomes are associated with V150 greater than approximately 50 cc and V200 greater than 22 cc in interstitial APBI patients. The SAVI dosimetry falls well within these guidelines based on our clinical experience with the device.^(11)^ We required the skin dose to be ≤100% of the prescribed dose and lung dose no more than 75% of the prescribed dose.

**Table 3:** Dosimetric guidelines for SAVI.

V90	>90%
V150	<50cc
V200	<20cc
Skin	<100% of prescribed dose
Lung	<75% of prescribed dose

### D. Patient selection

Initially, 21 patients who had undergone partial breast irradiation using the SAVI device were selected to retrospectively compare treatment modalities. Out of 21 total patients selected for our comparison, 38.1% were ineligible for the MammoSite balloon due to skin restrictions (skin bridge <7mm). Another patient was eliminated from the study as the modeling of the MammoSite in place of the SAVI was difficult and needed more advanced techniques (such as tissue deformation). Three patients eligible for both SAVI and MammoSite were eliminated from the comparison as they did not meet the NSABP B‐39 guidelines for 3D‐CRT. Finally, nine patients remained that were clinically eligible for all three treatment options. These nine patients were then replanned as both 3D‐CRT and MammoSite patients, and analyzed for comparison between modalities.

### E. Method of comparison

**Figure 2:**
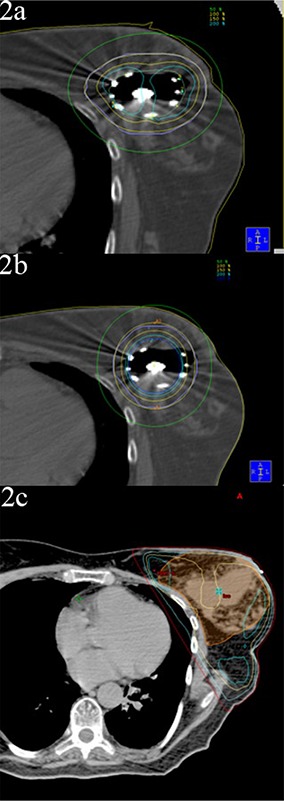
Single patient with 3 APBI modalities: a) SAVI device; b) MammoSite balloon simulated over the SAVI site; and c) 3D‐CRT plan (preimplant scan).

The CT scans from patients previously treated with SAVI (Fig. 2(a)) were planned with MammoSite by mimicking a balloon where the SAVI was located in the lumpectomy cavity (Fig. 2(b)). The surface of the balloon coincided with the outer struts of the SAVI and the minimum fill volume allowed for the MammoSite. A single dwell position was used in the center of the balloon and dose prescribed using Nucletron's Plato treatment planning software (Nucletron B.V., Veenendaal, Netherlands). CT scans of the patients (evaluation scans prior to SAVI implant) were then planned with 3D‐CRT (Fig. 2(c)) using Varian's Eclipse treatment planning system (Varian Medical Systems, Palo Alto, CA). Dose volume histograms (DVHs) were used to compare the treatment modalities as well as maximum point doses at the skin, lung and chestwall/rib interfaces.

## III. RESULTS

The results are shown in Table 4, where all values reported are the median of the maximum values. As can be seen in Table 4, coverage of the PTV (according to V90, where V90 is the fractional volume of target covered by 90% of the dose) was approximately comparable between each of the modalities with a range of 97.6%–100%. 3D‐CRT had the highest V90 coverage at 100%, while the SAVI and MammoSite were equal with V90 of 97.7% and 97.6%, respectively. V150 and V200 were nonexistent for the 3D‐CRT plans and were comparable between the SAVI and MammoSite.

**Table 4:** Dosimetric data (median of the maximum values) of nine patients studied comparing SAVI, MammoSite, and 3D‐CRT partial breast irradiation.

	*PTV Volume*	*V90*	*V150*	*V200*	*CW/rib*	*Lung*	*Skin*
SAVI	59.9 cc	97.7%	25.0 cc	10.4 cc	75.0%	64.7%	51.5%
MammoSite	71.5 cc	97.6%	23.9 cc	5.0 cc	100.0%	75.0%	95.0%
3D‐CRT	351.6 cc	100.0%	N/A	N/A	105.3%	93.8%	104.0%

## IV. DISCUSSION

The major difference between the plans was in the target volume treated, skin, lung, and chestwall/rib dose. 3D‐CRT clearly treats much more breast tissue, treating about five times as much volume as SAVI or MammoSite. SAVI had the lowest median of the maximum value of all nine patients for skin, lung, and chestwall/rib dose with values of 51.5%, 64.7%, and 75.0%, respectively. The 3D‐CRT plans had median values just larger than SAVI with values of 104.0%, 93.8%, and 105.3%, respectively. Likewise, the MammoSite plans had similar values to 3D‐CRT of 95.0%, 75.0%, and 100.0%, respectively. The single patient with the highest maximum values for skin dose had values of 75.0%, 108.6%, and 135.0% for the SAVI, 3D‐CRT, and MammoSite, respectively. Similarly, the single patient with the highest maximum dose to the lung had values of 80.0%, 104.9%, and 150.0% for SAVI, 3D‐CRT, and MammoSite, respectively. The highest maximum dose to the chestwall/rib was 100.0%, 114.7%, and 225.0% for SAVI, 3D‐CRT, and MammoSite, respectively.

The main limitation of this study is any tissue deformation or compression difference from mimicking a MammoSite in place of the SAVI. However, we used patients with generous skin spacing (minimum skin spacing for these nine patients was 1.0 cm and the median skin distance was 1.6 cm). Our analysis is a first order approximation in the comparison of modalities and highlights the main dosimetric differences, which we would not expect to change significantly with a more complex analysis. The SAVI is capable of contouring the dose to an asymmetric target volume, while the MammoSite cannot. Additionally, 3D‐CRT exposes a significantly increased amount of normal tissue because of the larger margins needed, due to the setup uncertainty from the high mobility of breast tissue and the entry and exit dose from the external beams.

## V. CONCLUSIONS

Three forms of APBI, MammoSite, 3D‐CRT and SAVI, were compared among nine patients. Target volume coverage was comparable between the devices; however, major differences are seen in the dose to normal tissue with the SAVI being the lowest, followed by the MammoSite and then 3D‐CRT. This indicates that the SAVI device is better able to adapt to each patient's geometry. Accelerated partial breast irradiation is becoming increasingly more attractive choice for women with early stage breast cancer and the SAVI device, with no skin or chestwall restrictions, may open the patient population able to choose APBI.
